# Typhus Abdominalis; Ganglianic Typhus; Nervous Fever

**Published:** 1844-05

**Authors:** 


ART. IV.—*Typhus Abdominalis; Ganglionic Typhus; Nervous Fever.* From Professor SCHOENLEIN's Lectures, published by some of his disciples. Translated by F. Roëlker, M.D., of Cincinnati.

The seat of this disease is in the abdominal organs, in the mucous membrane of the intestinal canal, and in the abdominal nervous system.

We discriminate three stages: 1. The stage of irritation; 2. The nervous, and 3. The critical stage.

A stadium prodromarum often precedes, and these premonitory symptoms are sometimes somewhat characteristic.—Weariness and heaviness in the limbs; sleep restless, not refreshing, interrupted with frightful dreams; loss of appetite; increased thirst, and frequent stools, mostly at night and seldom during the day. These symptoms often mislead physicians to the administration of irritating remedies, which only serve to hasten the development of the disease.

1. The stage of irritation. The beginning of the typhus diseased process may be dated from the time when the patient perceived the last chill, which was followed by permanent heat. We discriminate in this stage, (*a*) symptoms derived from the nervous system; (*b*) from the mucous membranes; (*c*) and from fever present.

(*a*.) Symptoms of the nervous system. The great weariness and atony, the leaden heaviness in the limbs and the dulness of the head increase. There is a deep sensation of sickness in the sensus communis, a reeling and staggering in walking; the eyes have a peculiarly faint and dull aspect, seldom real pain in the frontal and occipital region, but frequently starting up in sleep, rarely, however, delirium in this period.

(*b*.) Symptoms of the mucous membranes. The patients have a peculiar countenance, a gastric physiognomy, gastric color; the tongue is coated, whitish, slimy, and often bilious, yellowish, and filthy. Bitter, slimy taste, nausea, frequently amounts to actual vomiting. More or less pain in ileo-cæcal region, which, when it does not show itself immediately, often does appear after some motion. Constipation until the fourth day, then those characteristic stools occur, which consist of two parts, a superstanding liquid, as common intestinal mucus, colored yellow or greenish by a peculiar pigment; and of a subter-standing mass, which is flaky, and consists of albumen (which becomes less abundant in the blood), and of flocculi of a yellow color. The microscopical examination shows alkaline crystals, which are of an uncommon size and become visible at a twenty-fold magnification.

(*c.*) Symptoms of the fever. The symptoms of the general reaction may be of a sthenic, erethic, or of a torpid character. In that of the sthenic character there is violent chill, followed by heat, with calor mordax of the skin. The pulse is not particularly accelerated, but full and hard, from 80 to 90 beats in a minute. The tongue is covered with a white fur; thirst great; appetite bad. The fever is, however, more frequently of an erethic character. Shaking chill followed by permanent heat; skin rigid and dry; pulse accelerated, vigorous, 100 to 120 beats. The urine inconstant, often quite pale, mostly, however, dark-red and brownish. All these symptoms show a definite type of distinct exacerbations and remissions, the latter in the morning, the former in the evening. In the first stage the fever is seldom of a torpid character, in which the pulse is small and frequent, 110 to 120 beats, fluttering and easily compressible; subsultus tendinum, picking of the bed-clothes, &c.

*Complications.*—In this stage complications sometimes appear, especially violent affections of the brain, caused by strong congestions of it; and in this case violent furibund delirium takes place, commonly between the third and fourth day. The face is red, glowing hot; the eyes injected, protruded; the pulse full, hard, tense, 110 to 120 beats; tongue dry; thirst violent; carotids strongly pulsating; urine red. Encephalitis might be mistaken for it, which however is prevented by the characteristic diarrhœa, and the pains in the cæcum. Pneumonic symptoms often appear, especially in young, vigorous men, and at certain seasons. Sometimes oppression of the chest is felt, at other times a stinging pain; difficult respiration; cough; expectoration of mucus, which is sometimes streaked with blood; at some places dull sound upon percussion; and dry rhonchus, upon auscultation is heard.

*The duration of this stage* is seven days in its regular course, and in this case critical symptoms appear on the seventh or eighth day, which, however, disappear again; these commonly are, a moist skin, turbid urine, and epistaxis. Yet commonly on the evening of the eighth day, violent symptoms appear again, and the patient passes on to the second stage.

2. *The Nervous Stage.* Here we discriminate also: (*a.*) Symptoms of the nervous system; (*b.*) Of the mucous membrane; and (*c.*) Of the fever.

(*a.*) Nervous Symptoms. There is muscitating delirium from the beginning, which becomes more continued and eventually permanent. The susceptibility to external influences becomes gradually weaker; difficulty of hearing, and often deafness takes place. External irritations of the skin remain often a long time without effect. The patients lie motionless upon their back and mutter low to themselves; no loud talking is indulged in. The muscular strength is so much weakened, that they have constantly a tendency to slide down to the foot of the bed. The countenance altered, becomes stupid; the eye is dull, and without lustre. Also paralytical symptoms sometimes appear, as partial paralysis of the intestines and bladder; hence either involuntary evacuations of the fæces takes place, or torpor of the bowels exists; there is involuntary discharge of the urine, or retention of it, and as a consequence expansion of the bladder; and generally there is subsultus tendinum and picking of the bed-clothes; the patient constantly endeavors to remove things from his bed, which are not there.

(*b.*) Symptoms of the mucous membranes. The patient's tongue becomes dry towards evening; the base and superior surface are first affected,—the margins still remaining moist, but eventually the whole tongue becomes dry. It seldom remains clean, but frequently is covered with a thick, ropy, pitch-like slime. A similar coat is found on the teeth and mucous membrane of the nose, which always regenerates itself anew, as often as it may be removed. The abdomen becomes tympanitic, and the pains in the cæcum more intense, the stools copious, from ten to twelve within twenty-four hours, the most of which occur during the night; in the day-time they are moderate. The evacuations have the same quality as in the second division of the first seven days' period. Towards the eleventh or twelfth day, especially in pneumonic complications, cough developes itself, which soon becomes moist and rattling, yet is connected with little or no expectoration.

(*c.*) Febrile symptoms. The skin is burning hot, and either dry and husky, or becoming moist and dissolving in colliquative sweats, with a disagreeable odor. Ecchymoses frequently appear of herpes-like blots (false petechiæ), and of dark violet color, and especially are seen on the nates and back. Striped ecchymoses (vibices) are very bad symptoms, as they very soon ulcerate and are very apt to extend in width and depth. The pulse is small, thread-like, compressible, and empty; beats from 130 to 140 in a minute. The urine is muddy, increases in specific gravity, and reacts sour; the fæces, however, are of an alkaline nature, and thus a constant contrast shows itself between the secretions of the kidneys and those of the intestines.

The febrile symptoms commonly have a definite type of distinct exacerbations and remissions, these in the morning, those in the evening. In rare cases two exacerbations and remissions take place in a period of twenty-four hours, one remission being at noon, the other at midnight. Comparing however, the symptoms of several days together, an approximation to the tertian remittent type cannot be mistaken, for the observation presses itself upon us, that the intensity of the diseased symptoms always corresponds every other day, especially in typhus which arises from intermittents. In the second half of this second period, especially on the fourteenth day (sometimes earlier) critical symptoms appear—changes in the urine, with moisture of the skin; these symptoms are favorable.

*Complications*—with inflammation of the liver: typhus icterodes: rare in Germany, more frequent in southern Europe: The right hypochondrium becomes painful, swollen, and tense; jaundice developes itself.

3. *The Stage of Crisis.*—1. General crisis; the skin becomes moist, strong secretions appear, at first commonly in the evening only, but, by and by, the crisis continues longer, until the skin becomes continually soft and moist. If, however, the perspiration is to be a critical one, the pulse must become more quiet and the tongue moist, at least during its duration. The urine becomes clearer, and is discharged in greater quantity, sediments are seldom found in it.

2. Topical crisis. Instead of the former delirium, a quiet sleep ensues, after which the patients return again to consciousness as if awaking out of a long dream. The tongue becomes moist and the sooty coat of the teeth and of the mucous membrane of the nose begins to loosen itself. The cough becomes easy and the patients expectorate a phlegm like sputa cocta. The abdomen is soft, and the pains in the cæcum have disappeared. The exhausting diarrhœa subsides, and a constipation of two or three days' duration takes place. Although this favorable crisis appears, more or less violent disorders yet remain, depending upon ulcers present in the intestines, which mostly begin to heal in ten or fourteen days, but sometimes are protracted to the seventieth or eightieth day. Although convalescence is going on, the patients recover but slowly, and the following momenta are worth attention: 1st. The patients have an insatiable appetite, and hence are prone to eat too much, which acts very injuriously upon the healing of ulcers in the intestines. 2d. The sexual instinct awakens in a high degree; so as to produce frequent pollutions, nay often satyriasis.

*Varieties.*—1. As in measles, scarlet fever, &c., so we find here sometimes, though seldom, an abortive form. All symptoms of developing typhus are present, but on the fourth or seventh day, violent crisis through the skin or urine ensues, and thus the topical symptoms suddenly disappear. It would be desirable to be always able to produce this favorable termination.

2. Typhus abdominalis putridus—that is, abdominal typhus with symptoms of violent decomposition in the humors. Hæmorrhages of offensive decomposed blood ensue from the nose and rectum, hæmorrhage under the skin, vibices, ecchymosis, petechiæ. The urine is peculiarly alienated, more brown, putrifies sooner, and developes ammonia, or it shows decomposed blood, which appears as a red sediment. It has been tried to make another species out of this form, which however is improper, as a series of middle forms can be pointed out between simple abdominal typhus and putrid typhus.

3. Febris maligna sine febre. This may easily mislead the physician. The patients show only in the evening, between 9 and 10 o'clock, increased turgesence of the skin and excitement in the pulse; after midnight, these symptoms decrease again; and in the morning the skin is cool—nay, the extremities are very often completely cold, the pulse scarcely perceptible, and only towards midnight some vascular erethism appears. However, the topical symptoms of typhus are present. This condition, however, lasts but a certain time, and death mostly follows it; fever and delirium appear about thirty-six or forty-eight hours before its occurrence.

*Duration of the Disease*—Is in its regular course fourteen days. The crisis is between the fourteenth and fifteenth day, seldom on the ninth or eleventh, and if this is the case, mostly with fatal termination. The end is commonly also lethal when the crisis is delayed until the nineteenth or twenty-first day.

*Termination.*—1. *In perfect recovery,* distinct febrile crisis, which commonly holds the seven days' period.

*a.* General febrile crisis through skin and urine.

*b.* Topical crisis: uninterrupted sleep, expectoration of sputa cocta, and in typhus icterodes within twenty-four hours two or three black stools, of soft consistence and bad odor, which, however, makes the patient feel much easier.

Besides the increased appetite and sexual instinct, the patients often suffer in their convalescence slight difficulty of hearing, with ringing and humming in the ears. These symptoms are to be watched closely, for inflammation and suppuration of the internal ear are very apt to take place.

2. *In partial recovery.*—Various disorders remain. 1. Decubitus; it is formed from ecchymosis, and by no means from gangrene of the parts. It is often very extensive, so that the sacrum appears as if laid bare. Gangrene of external parts supervenes, as of the nose, the ears, or the big toe. When these parts become blue and livid, and lose their sensibility, gangrenous destruction is at hand. 2. Gangrenous furuncles, which often appear on the inner surface of the thigh, rapidly increase, and, upon lancing, secrete an ichorous liquid. 3. A certain kind of pseudo-erysipelas, one of the worst secondary disorders, which developes itself rapidly and as fast disappears again, upon which violent delirium, stupor, &c., ensue. 4. Parotitis: Violent humming and ringing in the ears, connected with a stinging pain in the neck along the course of the nervus vagus; the neck is very tender upon pressure, and the patient separates the jaws only with pain. A small, very painful tumor soon appears, which is at first movable, and often grows rapidly. Under improper treatment, these parotid swellings disappear, after which death commonly ensues. They never resolve without medical means; suppuration takes place, which by colliquation may bring on hectic phthisis, for the abscess grows rapidly downwards, and the carotid often in four days lies denuded at the bottom of it. 5. Suppuration in the internal ear: The humming and ringing of the ears are followed by a violent stinging pain in them—accompanied by vertigo, deafness, and under the symptoms of paralysis of the brain, death may take place. 6. Intestinal phthisis, when the ulcers in the mucous membrane of the intestines do not heal. It runs its course rapidly, and is accompanied by febris nervosa hectica. 7. Perforation of the intestine: It always takes place in the later periods only, mostly from the twenty-eighth to the sixty-fifth day, in the lower free part of the small intestine, but, according to Chomel, also in the free large intestine. The openings are always found at the bottom of an ulcer, where the muscular and serous coats are perforated. Their diagnosis is easy: sudden and extremely vehement pain in the abdomen, which swells, and becomes hard and tense; vomiting of massa herbacea; and facies hippocratica, and in consequence of the perforation and protrusion of the intestinal contents, inflammation of the peritoneum. 8. Secondary disorders in the lungs, either a simple blennorrhœa of the respiratory mucous membrane, or an inflammation in the upper lobes, or formation of tubercles. 9. Hydrops—one of the rarest secondary disorders.

3. In another disease. Intermittent fever is the only one which can develope itself, and this is but rare, and in those countries only where intermittent is endemic.

4. In death. It perhaps never ensues in the first period, but on the eleventh, fifteenth, seventeenth, or twenty-first days. The critical days may also be named fatal days. The uneven days are especially those which carry off the most patients. Death itself ensues in various ways: (*a*) in the height of the disease, by paralysis of the abdominal nervous system. The symptoms are, abdomen swelled, meteoristically tense, a peculiar pain produced by pressure upon the cæcum. The stools (from twelve to sixteen in twenty-four hours) often pass involuntarily, are thin, mixed with dissolved offensive blood. The countenance alters more and more, is covered with cold sweat, while the body feels constantly hot, the pulse is small, thread-like, worm-like, (puls. vermin.); singultus, subsultus tendinum come on. (*b.*) Through secondary disease. (*c.*) Parotitis developes itself and passes into destructive suppuration. (*d.*) In other cases hectic fever may come on. (*e.*) Excrescences form upon the intestinal mucous membrane; after the separation of the scab, a part of the mucous membrane is lost, which is not restored, but ulceration takes place and the patients die of abdominal phthisis. The symptoms in this case are the following: the diarrhœa continues, the peculiarly purulent masses appear in it, the patients have a permanent pain on the lower part of the small intestine, and the fever becomes hectic (violent heat towards evening, exhausting colliquative sweat until morning, an irritated frequent pulse). Where these symptoms appear, it generally terminates in fourteen days.

*Etiology.*—The disease is most frequent in the blooming years between the 18th and 26th; before puberty, and after the above mentioned period, it is rare. In old persons it is never found. Sex, also, seems to influence its frequency; for it is more frequent with women, especially young girls at the age of puberty, than with men. The proportion is from two to three. A peculiar distemper of the abdominal nervous system increases the susceptibility to the disease. Individuals, laboring under hysteria, epilepsy, or distemper of the ganglionic system from worms, are principally the subjects of attack.

*Cause.—External momenta.*—Exhaustion of the vital power through excessive exertion of the muscular action; also from the genital system through onanism and excess in coitus; through continuing diarrhœas, caused perhaps by misuse of evacuating remedies; through depressing passions; too long continued use of narcotics, (especially of cherry-laurel water and belladonna; as these remedies, besides their depressing influence upon the nervous system, at the same time induce decomposition of the blood.) If, under such circumstances, a general outbreak of the disease is to take place, a peculiar atmospheric constitution is required. Rapid changes in temperature, sudden transitions of weather, the decomposition of air impregnated with effluences from animal and vegetable matter. The predisposition to the disease increases under such circumstances to the utmost, if an individual, besides the influence of the nervous disease, at the same time is exposed to debilitating influences, such as bad nutriment; namely, the use of salted, half putrid meat. The disease may become contagious. The media of contagion are the exhalations of the patients; namely, the alvine evacuations, those from the skin, and perhaps the breath; hence this disease is frequently contracted by sleeping with or coming in contact with a person affected. On the other hand, however, a predisposition is always requisite.

*Anatomical Facts.*—1. Changes in the ganglionic system: If the patients die while the disease is at its acme, between the seventh and fourteenth days, the ganglia of the abdominal nervous system, especially the plexus solaris, are found to be swollen, enlarged, considerably engorged with blood, and consequently very much reddened. The redness, however, is not the florid one of inflammations, but it merges from purple red into livid; the same changes take place in the nerves, which connect the single ganglia. When death takes place somewhat later, the ganglia are likewise swollen, but their texture is harder, often of a fibro-cartilaginous nature, their color is whiter than in their healthy state. These changes have hitherto been overlooked. 2. Changes upon the abdominal mucous membranes: These have caused the assertion that abdominal typhus is identical with inflammation of the intestines; (namely, inflammation of the glands of Peyer); that this assertion, however, is invalid, is proved by merely observing the course of both diseases. In abdominal typhus, no symptom of inflammation is found until the fourth day of the disease; on the fourth day, symptoms appear that are totally absent in inflammation, which continue until the seventh day, at which time the nervous stage has developed itself. As regards the diarrhœa of abdominal typhus, we see that it is violent with one individual, while it is less so with another, and that, indeed, the intensity of the disease is in direct proportion to its violence. These changes in the intestinal function have of course a distinct diseased process for their foundation, not, however, the inflammatory, but an exanthematic one. The sectio cadaveris puts this fact beyond doubt. The exanthem has its appointed place in the lower half of the small intestine; does not easily pass over the valve of the colon into the cæcum, neither, however, passes upwards beyond the second third of the small intestine, and it is more diffused, the nearer it comes to the lower end of the intestine. In its simple course it forms at first a semi-globular nodule, which, later, on its top, gets a plate, or navel-like impression. This nodule is, in the beginning, covered with the intestinal mucous membrane, here after sometime an eschar is formed of grayish color merging into black, (as eschars are formed in carbuncle and anthrax), around which the margin of the nodule is elevated like a wall. Subsequently the eschar separates, which generally takes place from the tenth to the fourteenth day, and an ulcer is formed in its place, which in a regular course cicatrizes; in the opposite case, however, it continues and leads to abdominal phthisis. The nodules are of the size of a pea, and stand either singly or in groups, and then become confluent, as it is frequently found in small-pox. We now perceive, standing over the intestinal niveau, elevated surfaces full of breaks, of circumscribed form, and on their top, according to the number of confluent nodules, a corresponding number of eschars are seen. As to the manner in which the eruption developes itself, nothing is known with certainty. Whether, for instance, the eruption appears all at once between the fourth and seventh day, as in variola, (?) or whether additions are made during the nervous stage, perhaps towards the fourteenth day, as in varicella and varioloid, nothing is at this time decided. The probability, however, is that the eruption takes place within a definite period, between the fourth and seventh day, that the formation of eschars follows during the nervous stage, and that at the fourteenth day, either separation of the eschar and recovery or intestinal phthisis ensue. 3. Changes upon the respiratory mucous membranes: It is reddened from the bifurcation of the trachea into the minute bronchial tubes.

*Prognosis* is very unfavorable. One-third of the patients generally die. The proportion of mortality is worse than in the plague; of one hundred individuals thirty-three perish. It depends—1. On the causal momenta. Where the disease originated from exhaustion of the vital power, excess in coitus, continued use of purgatives, or narcotics, the prognosis is always worse than where atmospherical influences caused its generation. 2. On the stage. If the physician is called in the first stage, and acts properly, much can be done; if later, but little benefit can be expected. 3. On the regularity of its course. Where this exists, it is favorable; when the first stages are prolonged, it is very unfavorable. 4. On the intensity of the nervous symptoms. The more speedy the general collapse, the greater the alteration of the countenance—the worse is the prognosis. Twitching of the tendons, picking of the bed-clothes are very unfavorable. 5. The highest importance should be attached to stools. The earlier they appear, the more frequent they are, (three or four times in twenty-four hours is the rule); the more blood is mixed with them, and the darker and the more dissolute it is, the more we have to fear. Hæmorrhages are not absolutely fatal. 6. On the condition of the pulse. The more frequent it is, the more unfavorable, particularly if it loses at the same time its energy. 7. On the nature of the fever. The more distinct its remission, the more it approximates the type of tertiana, the more favorable it is. 8. The state of the respiratory organs. If these participate, the symptoms will not be so favorable, especially if it take place towards the sixth or seventh day; cough, if it occurs towards the eleventh day, is of less moment; its entire absence, however, is more favorable. 9. Appearance of the tongue. It is favorable when the margins and the tip of the simply dry tongue continue moist, and it retains its breadth; it is unfavorable, however, when the tongue becomes quite dry, more contracted, chip-like, and with stammering speech can utter no word distinctly. 10. Decubitus. The earlier it appears, the more unfavorable; still worse is the early appearance of vibices, especially when they rapidly grow. 11. Individuality. In very decrepid individuals it is unfavorable; also very plethoric individuals are very much in danger, on account of easily supervening apoplexy. 12. Crises. When they appear in due time, are harmonious and constant, they are favorable. Irregularity of the symptoms during the critical period is very bad. 13. Perforation of the intestine is absolutely lethal. 14. Affection of the breast, formation of tubercles, pseudo-erysipelas, make the prognosis very unfavorable. 15. In convalescence. We are to be led by the principle that the detection of unfavorable symptoms is of more importance in prognosis than a series of good ones.

*Treatment.—Prophylaxis.*—Since the contagion is fixed to the exhalations, sedes, &c., of the patients, we must endeavor, in order to avoid its transference, to decompose the exhalation and the alvine secretion as rapidly as possible. This is done by means of chlorine or pulverized coal, or fumigation of muriatic acid, in the vicinity of the patient; evaporation of alcohol and frequent renovation of air. For persons that must often be near the patient it is advisable to spit out their saliva, often to rinse their mouths with vinegar, to sleep in another room, to enjoy fresh air, observe the greatest cleanliness, and avoid all depressing influences.

*Treatment of the Disease itself.*—When symptoms of the received contagion appear, as nausea, headache, weariness, chilliness, an attempt may be made to remove it by means of an emetic. Tartariz. antim. may be administered, in combination with ipecac., in order to avoid its action upon the bowels, and an infusion of linden-blossoms or verbascum given for drink, and the patient advised to stay in bed. When the disease makes progress notwithstanding, it cannot be cut short, but will run through its stages. In rare cases where we get to treat the patients from the first beginning of the disease, it may happen that the disease perishes abortively on the fourth or seventh day, especially in forms with gastric symptoms, as nausea, inclination to vomiting, violent headache. Also, here an emetic may be given with advantage; but ipecac only should be used, to avoid laxative effects. In the other case, where these symptoms do not appear, the treatment has to be modified according to the various stages.

*First Stage of Irritation.*—The violent vascular irritation has to be moderated by suiting remedies, and where the disease is of a sthenic character, even venesection of 8 or 10 oz. may be necessary. Internally, we give the saline medicines, citrate of potassa either by itself or as effervescing draught, (potio Riverii); for drink, a weak lemonade or sugar-water. If, however, reaction is from the beginning of an erethic character, we give aqua oxymuriatica (chlorine) ℥j. in decoction of althæa. Constipation has to be removed by proper clysters; e. g. vinegar with some castor-oil. When pains in the cæcum appear, we must try to moderate them by frictions of ol. hyoscyam. coct. with unguent. hydrargyr. fortius. If they become very vehement, apply leeches, and afterwards emollient fomentations of linseed, with cicuta or herb. hyoscyam. To this comes a strictly antiphlogistic diet, gruel, barley-water, simple mucilaginous drinks—nothing irritating.

*Treatment of the Complications.* 1. The violent congestions towards the head with furibund delirium. Here general bleeding has been recommended; but it is advisable only where the fever is of a sthenic character; in the opposite case it certainly does more harm than good. The best is to apply sixteen or twenty leeches behind the ears, and cold fomentations to the shaven scalp. But where violent catarrhal affections exist, these fomentations have to be made of lukewarm vinegar and water; otherwise take snow and ice or Schmucker's fomentation.* Where these fomentations are not agreeable to the patient, they have to be stopped. Besides this, derivative remedies must be applied—sinapisms to the calves of the legs and soles of the feet, clysters of one-third vinegar and two-thirds decoction of bran. Internally give acids, muriatic acid ʒvi., to decot. althaeæ ℥vi.; sour drinks, lemonade, diluted sulphuric or phosphoric acid.

2. Pneumonic symptoms. Where symptoms of inflammation of the lungs exist, the treatment has to be directed against it. If the inflammation is confined to a small spot, topical bleeding of the inflamed part and lukewarm frictions

---

* Schmucker's cold fomentations consist of 4 lbs. water, 2 lbs. acetum vini., 1 oz. nitre, and 4 oz. muriate ammon.; or according to Richter, water lbs. 4, acetum vini lbs. 4, nitre oz. 16, muriate ammon. oz. 8. F. R.

of ol. hyoscyam, unguent. digitalis, and unguent. hydrarg. should be applied: if, however, the inflammation extends to a larger part of the lungs, and the fever is of a sthenic character, general bleeding besides topical is indicated. Internally mucilaginous remedies, emulsions, barley-water drink, and antiphlogistic diet.

If at the end of this first seven days' period, an attempt at a crisis should appear, which, however, is mostly but momentary, and rarely sufficient in degree, it must be properly supported; for instance, critical epistaxis is to be encouraged by the inhalation of warm vapors; crisis of the skin by means of pulv. Dover., infus. flor. verbasci or sambuci.

2. *The Nervous Stage.* When these momentary crises stop, and the disease passes over into the nervous stage, our whole attention should be directed to the specific diarrhœa, the accompanying fever, and the tendency to dissolution of the blood. Various remedies have been tried, tonics and astringents, logwood, rhatany, &c.; narcotics, especially opium, the preparations of iron, muriate of iron, as recommended by Autenrieth,* but none have proved satisfactory. The best effect was obtained from injections of starch with chlorine, and in the evening when delirium and restlessness appear, an addition of 20 or 30 drops of laudanum. Locally, when violent pains in the cæcum are present, make use of topical bleeding, and afterwards of fomentations of emollient herbs; but where there is a disposition to tympanitic swelling of the abdomen, apply irritating frictions of volatile liniment, with opium, linimentum saponis compositum. Internally, aqua oxymuriatica, (chlorine) either by itself in a mucilaginous vehicle, or according to circumstances, with an infu-

* Autenrieth gave murias ferri. gr. vi., xii., xvi., in twenty-four hours; Pammer gave from 5 to 10 grains of it every two hours in powder; an anonymous writer in Rust's Repert., gave tr. fer. mur. ʒiv. in aq., menth., pip., during the day, with the happiest effect. Vide Richter's Mat. Med., Vol. V. p. 64. F. R.

flor. arnicæ. Where symptoms of dissolution of the blood are present in a high degree, we administer the pure muriatic acid, ʒss. to ʒjs., or phosphoric and sulphuric acids in drinks. When in this stage bloody stools appear, the stronger astringents, as alum gr. x. to xx., with gum arabic are proper. Externally, the body should be sponged with vinegar, with diluted sulphuric acid, combined with whiskey or spirits of wild thyme.

The diet may be more nourishing in this stage; broth with the yolk of an egg; for drink, infus. flor. sambuci, light punch without lemon-juice; and in great debility, where pneumonic symptoms do not contraindicate it, wine, especially claret, but in small portions only, should be used, say three or four table-spoonfuls in a pint of decoction of barley or malt.

*Third stage of crisis.* Close attention should be paid in this stage to the crisis. Rarely is the eleventh day of importance, but more particularly so is the fourteenth, seventeenth or twenty-first day, as the crisis is apt to appear on one of these days. As soon as premonitory symptoms of the crisis appear, the whole attention of the physician must be directed to the production of a true or complete crisis. Individuals of a rigid, husky skin, have every evening to be placed in a bath of aromatic herbs, and warm water has to be poured over the free part of the body for five or ten minutes, then the patient has to be rubbed with a dry cloth and placed into a warm bed. Blisters may also be applied, but they are contraindicated when ecchymosis and decubitus are present. The effect of these external remedies must be supported by internal ones, as infus. valerian, with liquor ammoniæ acet., or liquor cornu cervi succin.;* further by small do-

* *Liquor cornu cervi. succinatus.*
℞ Acidi succini. ℥j.
solve in
Aquæ distillat. fervidæ, ℥viii.
solutioni immitte
Ammoniæ carbon. pyro-oleasi. q. s. ad perfectum saturationis gradum.
Filtra et serva.
Dosis: drops twenty or more. F. R.

ses of camphor, four or five grains in twenty-four hours in powder or mixture. Where the crisis is going on, warmer covering and a light aromatic tea are sufficient, instead of those remedies. The symptoms upon the intestinal mucous membrane commonly continue and must be counteracted by injections, (without opium, however), with the addition of eight or twelve drops of the liquid acetate of lead. To give this remedy by the mouth is not advisable, on account of its effects upon the stomach. When pains in the cæcum are present, when the mucous, cream-like evacuations again appear, when the pulse becomes strong, the antiphlogistic method before explained, has to be returned to.

In the treatment of the hectic fever, Peruvian bark is the principal remedy, in combination with mucilage where intestinal irritation is present; where such is not the case, with acids. Caution, however, is necessary, and the sweats must not suddenly be suppressed, for this can seldom be done without bad consequences. The diet should consist of easily digestible but nourishing victuals, broth, young meat, &c.; the drinks, of good old wine and water. The patient has to be watched in regard to his sexual instinct; for it must by no means be gratified.

*Treatment of some varieties.* Where there are symptoms of excessive dissolution of blood, as in febris putrida, the source of the colliquative hæmorrhage must be stopped as soon as possible. In hæmorrhage from the nose, cold fomentations may be applied to the root of the nose, and dossils o lint moistened with aq. vulneraria Thedenii* pushed into it. Where hæmorrhages under the skin, ecchymoses, vibices, petechiæ are existing, the surface has to be sponged with acet.

---

* Aqua vulneraria Thedenii.

℞ Aceti vini crudi, ℔siij.

Spirit. vini rectificati, ℔jss.

Acidi sulphurici, ℥j.

Aqua distillat. ℥v.

Mellis despumati, ℔j. Mix. F. R.

vin., with spirit. serpilli or whiskey—in urgent cases even with diluted sulphuric acid.

In complication with inflammation of the liver, topical bleeding, frictions with ung. hydrarg., and ol. hyoscyam. are indicated.

In the second stage, suppression of urine often takes place and is here a nervous symptom. The secretion of urine is either entirely wanting or is but scanty. The bladder has to be examined daily, and if necessary the catheter to be introduced; over the bladder unguent. camphor. and tinct. cantharid. may be rubbed in.

Of the secondary disorders we mention only the following five forms, as the others have to be treated according to general rules.

1. *Decubitus* is cured with more difficulty, than prevented in its development. The bed of the patient should be soft and smooth, the patient must be washed after each alvine evacuation with a sponge dipped into warm water, the parts lain upon must be washed with kirschwasser (a strong liquor) and lard, and the patient lie as much as possible on his side. After decubitus has developed itself, the ulcerating surface has to be dressed with unguent. saturn. with a small addition of pulv. opii; when the ulceration passes over into gangrene, it may be washed with aromatic infusions and decoct. cinchonæ. If the character of the ulcer is a mixed one, the treatment must be so likewise.*

6. *Gangrenous furuncles.* In the beginning they must be dressed with aromatic fomentations, and opened on the top

---

* Unguentum Autenriethii contra decubitum.

℞ Corticis quercus, part 1.

Aquae, part 8.

Coque ad dimidii partis remanentiam.

adde

Acetum Plumbi.

Quamdiu præcipitatum aritur, quod in colaterio charta emparetica tecto impositum cum aqua communi sedulo elotum in tantum siccetur ut pultem unguinosam præbeat, quæ loco frigida servanda est.

as soon as pus shows itself. Afterwards they have to be dressed with styrax ointment,* and the aromatic fomentations must be continued.

3. *Pseudo-erysipelas* generally terminates fatally; is treated with dry warmth, scarification, and over them aromatic fomentations perhaps might prove more successful.

4. *Parotitis* deserves our full attention on account of its danger. The following treatment has proved best. When a small painful tumor makes its first appearance, from eight to ten leeches must immediately be applied to the painful spot, and then frictions of ol. hyoscyam. If the tumor increases still afterwards, the topical bleeding should be repeated, and narcotic emollient fomentations applied. It should be opened as soon as possible.

5. *Inflammation of the internal ear.* A good number of leeches are to be put behind the ear, and after abscess has formed, we must endeavor to evacuate it externally as soon as possible. For this purpose we make emollient injections and pass vapors into the ear. After evacuation has taken place, put the patient in such a position as to promote its further discharge and keep the ear clean, and lastly, use gentle astringent injections.

---

* Unguentum de styrace. ℞ Styracis liquidi, elemi, ceræ flavæ aa ℥viiss. Colophanii ℥ij. Olei olivarum ℔sijss. Mix: fiat secundum artem unguentum.

